# Self-assessed vs. reported digital competence among health students in Germany, Ukraine and Kazakhstan: a DigComp 2.2–based cross-sectional study

**DOI:** 10.3389/frhs.2025.1673120

**Published:** 2025-11-26

**Authors:** Tom Schaal, Tim Tischendorf, Oksana Sydorenko, Makhabat Karagulova, Ruslan Chettykbayev, H.-Christian Brauweiler

**Affiliations:** 1 Faculty of Health and Healthcare Sciences, University of Applied Sciences Zwickau, Zwickau, Germany; 2 Emergency Medical Care, Horbachevskiy Ternopil National Medical University, Ternopil, Ukraine; 3 Department of International Affairs, International Higher School of Medicine, Bishkek, Kyrgyzstan; 4 Kazakh-American Free University, Ust-Kamenogorsk, Kazakhstan; 5 Faculty of Business and Economics, University of Applied Sciences Zwickau, Zwickau, Germany

**Keywords:** self-assessment, digital competence, health education, DigComp 2.2, cross-sectional study, students, Germany, Ukraine, Kazakhstan

## Abstract

**Introduction:**

Digital competence is essential for students and professionals in health and nursing education. Based on the DigComp 2.2 framework, this study examines the self-assessed digital competencies of students from Germany, Ukraine, and Kazakhstan across five core dimensions, aiming to identify national differences and potential misalignments between perceived and reported digital competences.

**Methods:**

A cross-sectional online survey (*n* = 269) was conducted among students in health-related fields. Participants rated their digital competence on 15 items aligned with DigKomp 2.2 questionnaire. Quantitative data were analyzed descriptively and with ANOVA (two-tailed, *p* < 0.05), using Games–Howell *post-hoc* tests in case of heterogeneity of variances and Kruskal–Wallis/Mann–Whitney tests as sensitivity analyses. In addition, an open-ended knowledge question asked respondents to describe their strategies for finding reliable online information. Responses were analyzed descriptively and qualitatively using inductive coding.

**Results:**

While all groups reported generally high digital competence, German students rated themselves significantly lower in the Digital content creation dimension compared to their peers and the KaWuM reference sample. However, their responses to the open-ended question revealed methodologically advanced search strategies, including systematic literature reviews (*n* = 8), Boolean operators (*n* = 6), and use of AI tools (*n* = 1). Ukrainian students emphasized heuristic and comparative approaches, while Kazakhstani responses reflected pragmatic strategies under infrastructural constraints.

**Discussion:**

The findings suggest a mismatch between self-assessed and actual digital competence, particularly among German students, who may underestimate their skills. This highlights the importance of triangulating quantitative self-reports with qualitative diagnostics. The study underscores the need for embedded digital skills training, especially in Digital content creation, across national contexts in health education.

## Background

Digitalization in healthcare and nursing contexts is an irreversible development, but approaches in the healthcare sector and among supporting professions vary greatly and have so far been classified in a largely unstructured manner, both nationally and internationally (1). In addition to robotic systems, information and communication technologies, digital monitoring, process support, and artificial intelligence (AI) are already being used and will continue to gain importance in the future. The skills of healthcare professionals are crucial for the proper use of digital technologies, taking into account roles, responsibilities, and ethical and professional dilemmas (2).

**Figure 1:**
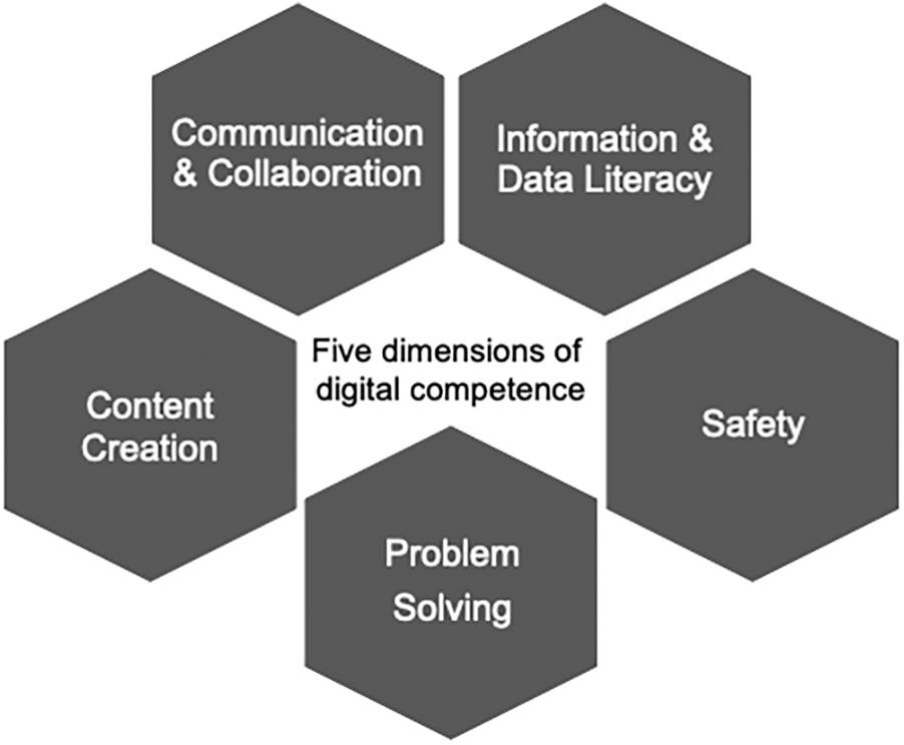
The five dimensions of digital competence, adapted from (3) (DigComp 2.2 framework, European commission, JRC).

Regarding the definition of the European Union digital competences involve the confident and critical use of digital technologies, which are used for information, communication and problem-solving strategies in all areas of life (3). Alongside other basic skills such as reading, arithmetic and writing, the European Union (EU) defines digital competence as one of the eight key competencies for lifelong learning (Figure 1) (4). Due to its broad scope, adaptability, and policy relevance, the European framework DigComp 2.2 has emerged as one of the most widely used reference models for digital competence development across sectors, including health and education (5). In this study we measure digital competence using DigKomp 2.2 questionnaire (6), an instrument aligned with DigComp 2.1 descriptors. We frame results within the EU DigComp family of frameworks and reference DigComp 2.2 for up-to-date examples, while not claiming a change in the measured construct. Beyond digital competence and engagement, recent scholarship emphasizes the integration of sustainability-oriented “green” digital skills. The GEARS framework (Goals, Enablers, Activators, Recognition, Solutions) provides a useful lens for designing digital learning that combines technical proficiency with environmentally responsible and ethical practice (7).

It should therefore be noted that digital competence should be taught in practical settings during training and continuing professional development programs for healthcare and nursing professionals, in the spirit of lifelong learning—especially in light of future developments and opportunities in digitalization and the increasing use of AI (2).

In terms of international comparison, Germany is in 13th place in the European ranking of the digital economy and society in 2022. Leading the way are the Scandinavian countries Finland and Denmark (8). In a survey conducted in May and June 2021 by the ifo Institute (Leibniz Institute for Economic Research), 56 percent of respondents in Germany at the time of the survey said that digital and media skills were very important for the future of society. A further 36 percent rated them as rather important. Looking beyond the European Union, considerable heterogeneity in the digital transformation of health and care systems can be seen internationally. Although European frameworks such as DigComp 2.1 and the updated DigComp 2.2 emphasize the importance of digital competence for societal participation and lifelong learning, the extent to which digital infrastructures and services are implemented and used varies considerably between countries (9). In Ukraine, the national eHealth system has undergone significant development since 2017. It enables the use of electronic prescriptions, medical records, and digital patient identification, supported by centralized registries and evolving legal frameworks. While standardization and interoperability remain work in progress, an estimated 40%–50% of the population may already be engaged with digital health services (9, 10). Kazakhstan has also made substantial progress in developing a digital health ecosystem that aligns with international standards. Since 2013, the country has developed a comprehensive digital health strategy coordinated by the Ministry of Health. This strategy aims to establish patient-centered care and strengthen data exchange between service providers. Implementing HL7 FHIR standards—a modern, web-based interoperability framework that enables efficient and standardized exchange of healthcare information—and regional health information systems demonstrates a commitment to structured digitalization, with an estimated 30-40% user engagement. Nevertheless, data fragmentation and uneven implementation across regions continue to pose challenges (9, 11). In contrast, Germany exhibits a mature institutional and regulatory framework for digital health, with legal mandates supporting the use of the electronic health record, the electronic prescription, and participation in the national telematics infrastructure. However, implementation remains fragmented. Despite their legal availability, less than one in five insured individuals actively uses digital health services (12). Reasons for this gap include regulatory complexity, data protection concerns, lack of interoperability across IT systems, and limited incentives for providers. The WHO characterizes Germany's digital health system as advanced in structure but delayed in practice (9). This international comparison highlights the need to complement infrastructure with human capacity by fostering digital competence among healthcare professionals, which is a prerequisite for sustainable digital transformation.

Current educational studies warn against neglecting digital competences among the generation of digital natives. The fact that a person has grown up surrounded by digital applications does not automatically make them digitally competent. Their informally acquired skills are incomplete. A distinction must be made between digital lifestyle competence and digital workplace competence. Formal training is essential, especially for the latter, in order to be able to use digital applications efficiently, safely, and competently. The education sector must therefore see itself as a mediator in this area of competence development in order to transfer digital lifestyle competences into digital workplace competences and prepare people as well as possible for the digital transformation process (13, 14).

To illustrate the relevance of digital competences, the global megatrends should not go unmentioned. In this context, they include globalization, artificial intelligence, health and, finally, digitalization. To be able to act efficiently and to participate in international comparisons and competition, digital competencies are among the essential areas of competence in dealing with such far-reaching changes as the aforementioned megatrends (15). Current evidence suggests that the mode of delivery—online or face-to-face—does not inherently determine learning success when design quality and learner support are comparable. Recent large-scale findings indicate broadly equivalent engagement and satisfaction outcomes across modes, emphasising the importance of contextual and pedagogical factors over the delivery format itself (16).

Building on the results of the KaWuM study, which identified career paths and qualification requirements in science and higher education management using the DigKomp 2.2 questionnaire in Germany, this study aims to show how digital competences are assessed among students in Ukraine, Kazakhstan, and Germany. The following research questions can be derived: RQ 1—To what extent do students in Germany, Ukraine, and Kazakhstan differ in their self-assessment of their digital competence in the various dimensions of DigComp 2.2? RQ 2—To what extent do the self-assessments of digital competence correspond to the complexity of the search strategies indicated in the open-ended responses? Current research increasingly uses performance-based or rubric-based observation methods to supplement self-assessed digital competence. Seifert & Lindmeier (17) developed a performance-based assessment in mathematics teacher training that revealed mixed relationships between self-assessment and actual task performance (17). Similarly, González-Mujico et al. (18) validated rubric-based frameworks that integrate observation and task performance alongside self-assessment, revealing significant gaps between perceived and demonstrated competence (18). Taking these findings into account, the chosen mixed approach of quantitative self-assessment combined with qualitative open-ended responses describing the sophistication of the search strategy is justified (6). The participating students come from the fields of health management, nursing, and medicine. The DigKomp 2.2 questionnaire served as the basis for capturing digital literacy characteristics in accordance with the EU reference framework (6).

## Methods

### Questionnaire

Against the background of globalization and networking of researchers from different countries worldwide, it is particularly important to survey digital competencies with the help of a uniform construct. The DigKomp 2.2 questionnaire according to Krempkow (6) represents a theoretically and empirically based survey instrument for recording digital competencies according to the DigComp2.1 reference framework of the EU (19), which was used in the Germany-wide KaWuM Survey 2 in 2022 (6). The DigKomp 2.2 questionnaire captures with 15 items all five dimensions (subscales) of digital competencies according to DigComp2.1. The subscales are (1) *Data processing & evaluation*, (2) *Communication/Collaboration*, (3) Digital content creation, (4) Security, and (5) Problem solving. Responses are given on a five-point scale ranging from 1 (not at all) to 5 (to a very great extent) (6). Internal consistency was assessed by calculating Cronbach's alpha for each of the five DigComp 2.2 dimensions at both the country level and for the overall sample. In addition, an open-ended knowledge test question is asked to capture the magnitude of, in some cases, suspected overconfidence in data processing/assessment. The open-text responses were analyzed by two researchers with backgrounds in health education and digital competence. Both coders first independently read all responses to gain familiarity with the data. We then developed an initial set of categories inductively based on recurring patterns (e.g., systematic, heuristic, pragmatic strategies). These categories were discussed and refined collaboratively until consensus was reached, forming a codebook. All responses were subsequently coded manually in Excel. A detailed codebook containing category definitions, inclusion and exclusion rules, anchor quotes, and code frequencies by country is provided in the Supplement to ensure transparency and reproducibility. A potential overestimation of the reliability of internet-based research strategies can be avoided by systematically analyzing and triangulating results across multiple sources. This approach provides a broader, more balanced perspective, contributing to a more comprehensive and critical assessment of the subject matter. The questionnaire was administered exclusively in English across all three countries.

### Duration and target group

Invitations to participate in the online survey were distributed via local contact persons at the participating universities, who circulated the link through institutional mailing lists. The questionnaire was hosted on SoSci-Survey and accessed 758 times, of which 269 responses were fully completed. Only students enrolled in health and nursing sciences or related courses were eligible. Incomplete responses were excluded. No incentives were offered for participation. On the landing page of the online survey, participants had to actively agree to the terms and conditions by clicking a button before accessing the actual questionnaire. Otherwise, they were redirected to the end of the survey. The survey was conducted in compliance with applicable data protection regulations, with a comprehensive data protection concept available on the landing page. The survey period was from December 2024 to April 2025, with a reminder email sent in February. The participating institutions were the West Saxon University of Applied Sciences Zwickau (Germany), Ivan Horbachevsky Ternopil National Medical University (Ukraine), and Kazakh-American Free University (Kazakhstan).

### Statistical analysis

All statistical analyses were performed using IBM Statistics SPSS version 30 for Mac. Both descriptive and inferential statistics were conducted. Statistical significance was determined at a two-tailed alpha level of *p* < 0.05. Prior to conducting inferential analyses, the distribution of the variables was assessed for normality using the Kolmogorov–Smirnov test, and homogeneity of variances was evaluated using Levene's test. For group comparisons, analyses of variance (ANOVA) were employed. In cases where Levene's test indicated heterogeneity of variances, the Games-Howell *post-hoc* test was applied. The Games-Howell test does not require p-value correction, as this is done automatically by adjusting the degrees of freedom. Even though simulation studies demonstrate the robustness of ANOVA with regard to violations of the normality assumption, for non-normally distributed variables, the Kruskal–Wallis test was calculated as a sensitivity analysis and the Mann–Whitney test with Bonferroni correction was calculated as a *post hoc* test (20, 21).

## Results

The survey was conducted among a total of 269 students. The sample consisted of 49 German students (*n* = 8 male; *n* = 40 female; *n* = 0 diverse; *n* = 1 no information) and 55 Kazakh students (*n* = 13 male; *n* = 40 female; *n* = 0 diverse; *n* = 2 no information) and 165 Ukrainian students (*n* = 43 male; *n* = 120 female; *n* = 2 diverse; *n* = 0 no information). Cronbach's alpha coefficients were calculated for each of the five dimensions both at the country level and for the overall sample (Table 1).

**Table 1:** Internal consistency (Cronbach's α) of the five DigKomp 2.2 dimensions across countries and for the total sample.

Dimension	Country (Cronbach's alpha coefficient)			Total
	Kazakhstan	Ukraine	Germany	
Data processing & evaluation	0.84	0.88	0.88	0.87
Communication/Cooperation	0.78	0.72	0.59	0.72
Digital content creation	0.80	0.79	0.80	0.80
Security	0.76	0.90	0.80	0.84
Problem solving	0.75	0.86	0.78	0.82

Across all countries and dimensions, values exceeded 0.7, and in several cases, were above 0.8, indicating acceptable to good internal consistency. Specifically, coefficients greater than 0.7 are considered acceptable to good, while those above 0.8 are regarded as good. The only exception was observed in dimension 2 for Germany, where the Cronbach's alpha did not reach the threshold of 0.7. It is noteworthy that only fully completed questionnaires were included in the analyses, as the online survey required all items to be answered prior to submission. This finding suggests that the employed measurement instruments exhibit a high degree of reliability in each national context, thereby supporting the validity of cross-cultural comparisons. The comparable Alpha values confirm that the scales function consistently and adequately regardless of cultural differences, enabling meaningful interpretation of results. Consequently, the measurement instruments used in this study are both culturally equivalent and psychometrically robust, facilitating valid comparisons of constructs and ensuring that observed differences in responses can be reliably attributed to substantive factors rather than methodological inconsistencies.

**Figure 2:**
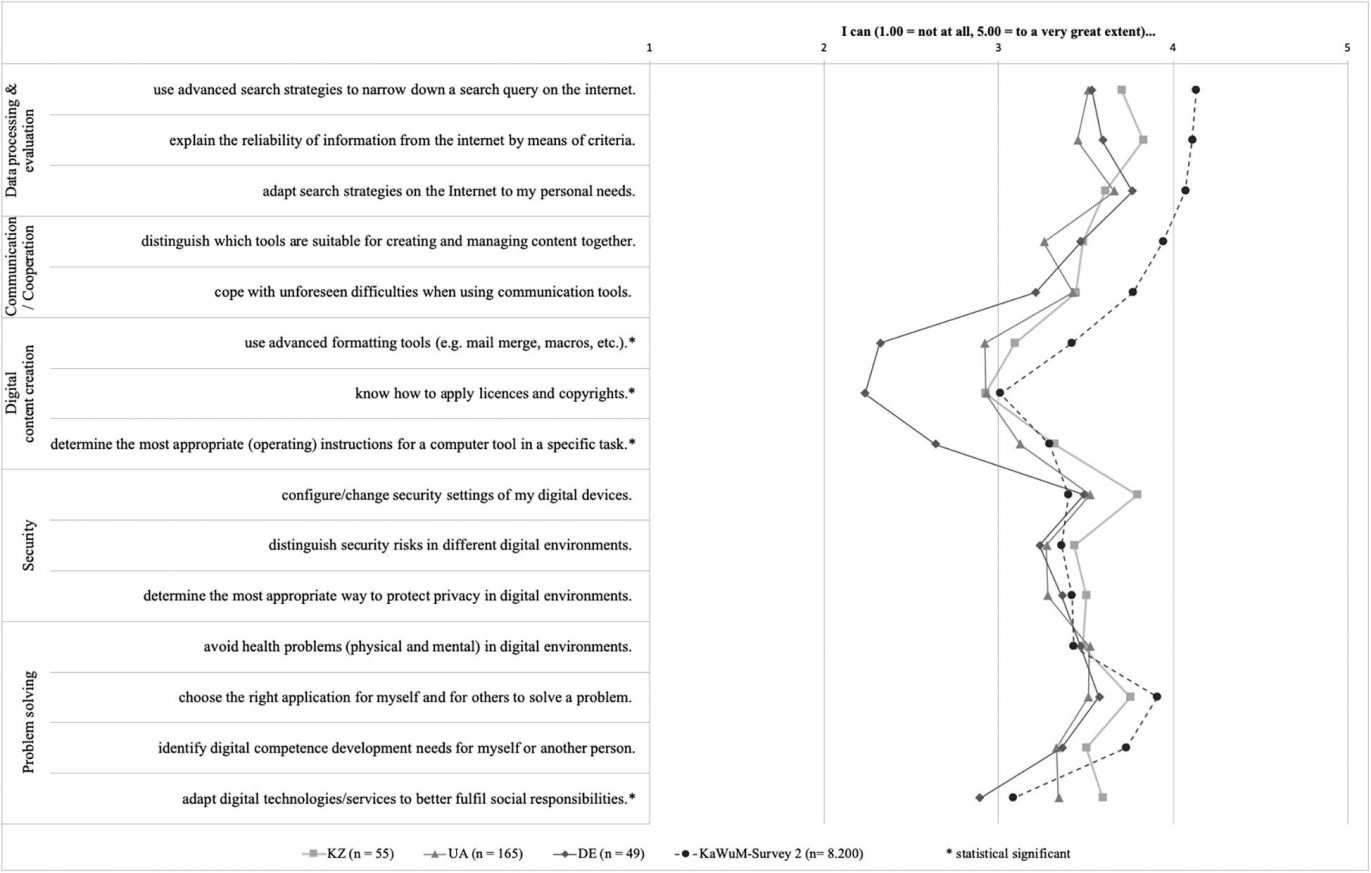
Mean values of the digital competencies recorded (*difference statistically significant). Note. 1.00 = not at all, 5.00 = to a very great extent.

The digital competence related variables were not normally distributed and exhibited heterogeneous variances. Overall, differences in mean values of digital competencies were significant for 4 out of 15 items (Figure 2). The KaWuM Survey 2 values are provided for orientation only and were not incorporated into the statistical analysis, as the cross-national comparison was primarily exploratory and only mean values were available for the KaWuM Survey 2 measures, which precluded the use of ANOVA. Significant differences were observed for the items: “I can use advanced formatting tools (e.g., mail merge, macros, etc.)”, “I know how to apply licenses and copyrights”, “I can determine the most appropriate (operating) instructions for a computer tool in a specific task” and “I can adapt digital technologies/services to better fulfill social responsibilities”. These significant differences existed between Ukraine and Kazakhstan on the one hand and Germany on the other, but not between the two post-Soviet countries. Exact p-values, confidence intervals, effect sizes and standard errors are provided in the e-Supplementary Material, along with the dataset for download (e-Supplementary Material 1). Due to the lack of normal distribution, a sensitivity analysis was performed using the Kruskal–Wallis test and subsequent Mann–Whitney test (Bonferroni correction). There were no deviations from the ANOVA in terms of significance. The item-related means by country and overall, as well as pooled SD and Cohen's d and the p-values of the sensitivity analysis, are also available for download (e-Supplementary Material 2).The data set has also been uploaded to a freely accessible repository (25).

Additionally, an open-ended knowledge question related to the first dimension (*Data processing & evaluation*) asked respondents what strategies they use to find reliable information online. Responses from Germany (*n* = 18), Ukraine (*n* = 16) and Kazakhstan (*n* = 2) revealed both shared patterns and country-specific nuances. Participants from Germany described highly systematic and academically rigorous strategies. The most frequently mentioned approach was conducting systematic literature reviews (*n* = 8), often guided by frameworks such as RefHunter or the PICO scheme. As one student put it: “Systematic literature search (10 steps according to RefHunter)” (DE-98). Boolean operators (AND, OR, NOT) were commonly used in databases such as PubMed and Google Scholar (*n* = 6), with responses such as “Keywords, key terms, Boolean operators (AND/OR/NOT)” (DE-112). The snowball method of tracing citations from relevant articles was also employed (*n* = 4). Other recurring strategies included using scientific databases and official sources, such as national statistics portals, and formulating search strings using keywords, synonyms, and wildcards. Some participants also mentioned using ChatGPT to generate keywords (*n* = 1) and reflected on the growing role of AI tools in replacing manual search processes (*n* = 1).

Ukrainian respondents reported a reflective approach that was slightly more heuristic. They emphasized the importance of critically evaluating and comparing Ukrainian and international sources (*n* = 3), as well as precise wording and clearly defined search queries (*n* = 3). The use of professional and academically recognized resources was also reported (*n* = 2). Advanced techniques such as semantic searches, truncated or wildcard terms, and keyword optimization were also mentioned. Furthermore, several participants referred to consulting evidence-based literature, medical protocols and European clinical guidelines (*n* = 2). One respondent also noted the use of neural networks and browser-based tools (*n* = 1). Illustrative quotes included, for example: “Identify key concepts—find a few different resources—compare them with each other” (UA-374), and “Medical protocols, research articles, and specialized literature are my main references” (UA-658).

In contrast, the responses from Kazakhstan were more concise, but indicated flexible and adaptable search behavior. It should be noted at this point that, due to the small number of free-text responses, there is a qualitative sample imbalance and these can only be considered illustrative, with generalizations and comparisons being severely limited. One participant simply mentioned “OSINT” (KZ-348), pointing to the use of open-source intelligence techniques. Another described starting broadly and then narrowing the search if the first attempt was unsuccessful: “Firstly say something related to the search and if I don’t find anything, I narrow it down” (KZ-491). Although less technical than the German or Ukrainian examples, these responses reflect a pragmatic approach that may be influenced by different academic norms or access to resources.

In summary, although all respondents emphasized the importance of reliable sources and keyword-based search strategies, the German participants reported the most structured and technical methods, the Ukrainian participants combined analytical reasoning with modern tools, and the Kazakhstani participants relied on intuitive and flexible approaches. These differences suggest that search competence is shaped not only by individual skill, but also by institutional practices and digital infrastructures.

In addition, mean values were calculated and tabulated for each of the five dimensions of digital competence according to DigKomp 2.2 questionnaire for further comparison (Table 2).

**Table 2:** Overview of mean values per dimension of digital competencies.

Dimension	Country (mean value)		
	Kazakhstan	Ukraine	Germany
Data processing & evaluation	3.75	3.54	3.63
Communication/Cooperation	3.46	3.34	3.34
Digital content creation	3.09	2.98	2.40
Security	3.61	3.36	3.36
Problem solving	3.75	3.54	3.63

Note. 1 = not at all, 5 = to a very great extent.

## Discussion

The results of the present study offer important insights into the self-perceived digital competencies of students in Germany, Ukraine and Kazakhstan across the five dimensions of the DigComp 2.2 framework. While overall competence levels appear relatively high, a differentiated analysis reveals both statistically significant group differences and potential mismatches between self-perception and actual skillsets. Of the nine significant characteristics, five had a medium effect (0.3 < d ≤ 0.5) and four had a large effect (0.5 < d ≤ 0.8) (22). The largest effect was found in “I know how to apply licenses and copyrights” between Germany and Ukraine (d = 0.63) (e-Supplementary Material 2).

Notably, the dimension of Digital content creation stands out, as it revealed statistically significant differences between the German sample and the other two student groups, as well as the KaWuM reference sample. German respondents reported significantly lower self-assessments across all three items related to Digital content creation (e.g., applying licenses and copyrights, determining appropriate operating instructions), suggesting a comparative deficit in this critical area. These findings mirror previous research highlighting the need for structured support and formalized training in digital content-related competencies, particularly in health-related education contexts (23, 24). In Germany, the problem of insufficiently developed digital skills in the health and education sectors has been recognized (1, 2, 8). The DigiK-Part project addressed this deficit by developing tailored training and continuing education modules that are explicitly based on the European reference framework DigComp 2.2. Accompanying surveys showed that the level of digital skills in the countries used for comparison is relatively similar, with the exception of dimension 3, “Digital content creation”. This reveals potential synergies for adapting the modules developed to other target regions, in particular Ukraine and Kazakhstan. This also represents a recommended starting point for further research activities. Against the backdrop of the comparable starting point, microcredential programs in particular can create added value for the post-Soviet countries, as no significant differences in skill levels between the countries could be identified. Given the comparable starting levels, our findings suggest potential benefits but do not permit generalization beyond the three institutions studied. Our results align with recent evidence showing that well-structured online formats can achieve comparable engagement and satisfaction to face-to-face teaching when supported by deliberate design and feedback mechanisms. This reinforces the importance of focusing on instructional quality and learner support rather than delivery mode alone (16).

To further validate and contextualize these self-assessments, an open-ended knowledge question was included, asking participants to describe their personal search strategies for locating reliable information online. This question, linked to the first dimension of *Data processing & evaluation*, aimed to reveal potential discrepancies between stated and actual digital competence levels—particularly with respect to evaluative and information-seeking skills. The open-ended responses capture self-reported strategies and should not be interpreted as performance testing.

Ukrainian participants appeared more heuristic in their reasoning, emphasizing accuracy and critical source comparison, while Kazakhstani responses reflected flexible and adaptive thinking. These differences likely reflect not only individual preferences, but also institutional training cultures and digital infrastructure disparities.

Interestingly, a discrepancy emerged between self-assessed and reported digital competence, particularly in the German sample. While German respondents rated their skills relatively low in the closed-ended items, their open-text responses revealed sophisticated academic search strategies, including systematic literature reviews, Boolean logic, and citation tracing. This contrast highlights the limitations of self-assessment instruments in capturing actual capabilities. This echoes findings from Krempkow et al. (13), who observed a frequent discrepancy between the perceived and actual digital skills of students (13). Conversely, brief or general responses (e.g., “Google”) in other samples may reflect an overestimation of skills in the absence of structured training or standardized criteria (23). Cross-cultural comparability requires careful consideration. Although subscale reliabilities were broadly comparable across countries, suggesting a tentative degree of functional equivalence, true measurement equivalence cannot be assumed. Items may be understood differently depending on language proficiency, educational conventions, or access to digital infrastructures, raising the possibility of differential item functioning. Observed group differences should therefore be interpreted within the broader contextual descriptions of the digital health ecosystems in Germany, Ukraine, and Kazakhstan, but without implying causal attributions to system-level factors. We consider these analyses exploratory and hypothesis-generating, providing a foundation for future research rather than definitive conclusions.

Taken together, the findings underscore the complex interplay between infrastructure, educational context and individual competence development. They also suggest that the cultivation of digital skills—especially in evidence-based information practices—requires not only technical access and institutional support, but also formal training opportunities. These should begin during undergraduate education and extend into professional development initiatives, ideally embedded in national qualification frameworks and EU-level standards such as DigComp 2.2 (5). Based on our findings, we recommend embedding targeted micro-modules into health curricula that address specific areas of digital competence where gaps or opportunities were identified. For DigComp 2.2 dimension Digital content creation (3), German students reported lower self-assessments compared to their peers, which suggests the need for modules on the practical application of licensing and copyright principles, the use of advanced document formatting and automation tools such as mail merge and macros, the development of multimodal content standards for patient education and interprofessional communication, and the basics of data stewardship, including version control, metadata, and secure sharing. For DigComp 2.2 dimension information and data literacy (3), the sophisticated strategies reported by many German participants highlight the value of teaching evidence-based search techniques more broadly and earlier in training. This should include Boolean logic, systematic database selection, citation chaining, and foundational prompt engineering for AI-assisted search, with consistent emphasis on source verification and quality assurance. Each of these micro-modules can be aligned with the relevant DigComp proficiency descriptors [for example, “Applying copyright and licences” (3.2), “Developing digital content” (3.1), “Browsing, searching and filtering data” (1.1), “Evaluating data” (1.2)] (3), which enhances their recognition and portability across institutions and systems. Finally, the design of these modules should take into account infrastructural constraints by, for example, offering offline learning materials or focusing on freely available repositories in contexts with limited digital resources, thereby ensuring their adaptability to the different realities of Germany, Ukraine, and Kazakhstan. In line with this broader understanding of digital competence, future micro-modules could draw on the GEARS framework to foster both digital and sustainable practices—for example, responsible data use, low-energy content workflows, and ethical AI prompting—without extending beyond the current study's empirical focus (7).

In conclusion this study provides insights into the digital skills of students in the health sector in Germany, Ukraine, and Kazakhstan. Quantitatively, differences between the countries were evident, particularly in the area of digital content creation, where German students reported comparatively lower self-assessments. Qualitative results complemented these findings by showing that German students employed highly systematic and academically sound strategies, while students from Ukraine and Kazakhstan tended to use more heuristic and pragmatic strategies for information retrieval. Future research should expand on these findings by incorporating performance-based tasks to validate self-assessments, testing measurement invariance across languages and contexts, and evaluating targeted training modules such as micro-credentials or digital literacy programs. Such approaches can help clarify the relationship between self-perceived and demonstrated competencies and develop evidence-based educational strategies.

## Limitations

Several methodological limitations should be acknowledged. One limitation of our study is that the link to the survey was not personalized, so we were unable to systematically check for potential duplicate entries. Although we did not find any anomalies in response patterns or completion times that would indicate multiple submissions by the same person, the possibility of duplicate participation cannot be completely ruled out. This cross-sectional study uses convenience samples from three countries and relies on self-assessments without behavioral performance tasks. The tests at the item level are exploratory and subject to multiplicity. Primary conclusions are drawn at the subscale level with FDR control. Another limitation of this study concerns the unequal sample sizes across countries. Statistical power is reduced in groups with smaller samples, such as Germany (*n* = 49) and Kazakhstan (*n* = 55), making it possible that real differences between groups or individual items may not reach statistical significance. Additionally, smaller sample sizes result in wider confidence intervals and less precise estimates, particularly in item-level analyses. This imbalance may have led to a failure to accurately detect significant differences at the item level. In contrast, the larger sample from Ukraine (*n* = 165) allows for more reliable item-level comparisons, while random effects and variance may have a greater influence in the smaller country samples. These factors should be considered when interpreting the results. Due to its small size, the qualitative Kazakh sub-sample (*n* = 2) precludes comparative statements regarding the free-text responses and serves only as an illustration. No formal tests of measure invariance across languages were conducted. Future work should supplement performance-based tasks, test (partial) invariance, and increase the qualitative subsamples across all locations. Personalized survey links were not used, as such links would have become invalid in the event of internal forwarding within institutions. As a result, technical exclusion of multiple participations was not possible. The questionnaire category on digital competences was based on the theoretically and empirically grounded DigKomp 2.2 questionnaire (6). However, it must be noted that the self-assessment format inherently entails the risk of bias, such as over- or underestimation of one's own competences. This should be considered when interpreting the quantitative results. Furthermore, the exclusively English-language administration of the online questionnaire may have led to language-related biases among some participants. Finally, the dropout rate during the survey may have been influenced by factors such as social desirability bias or survey fatigue, which are common challenges in voluntary and self-administered online questionnaires.

## Data Availability

The original contributions presented in the study are included in the article/Supplementary Material, further inquiries can be directed to the corresponding author.
