# Supplementary material for: Self-assessed vs. reported digital competence among health students in Germany, Ukraine and Kazakhstan: a DigComp 2.2–based cross-sectional study
Source: Front Health Serv. 2025 Nov 26;5:1673120. doi: 10.3389/frhs.2025.1673120 (PMC12689590; doi:10.3389/frhs.2025.1673120)
Supplement: Supplementary file 2 [file Datasheet2.pdf]

Codebook qualitative analyses.

Code	Definition	Exclusion (not included)	Anchor Quotes (anonymized)	n (DE)	n (UA)	n (KZ)	n (Total)
**Systematic Literature Review**	Structured, multi-step searches, often based on RefHunter or PICO schemes.	General keyword or ad hoc searches.	“Systematic literature search (10 steps according to RefHunter)” (DE-98)	8	0	0	8
**Boolean / Search Operators**	Use of Boolean operators (AND, OR, NOT), search strings, wildcards.	Database use without logical operators.	“Keywords, key terms, Boolean operators (AND/OR/NOT)” (DE-112)	6	0	0	6
**Snowball / Citation Chaining**	Following references from relevant articles to find additional sources.	Keyword expansion without tracing references.	“Snowball method for free search” (DE-121)	4	0	0	4
**Databases / Official Sources**	Use of academic databases (PubMed, Google Scholar) or official resources (e.g., national statistics portals).	Non-academic general web search.	“Use of scientific databases and official sources such as national statistics portals” (DE-205)	5	0	0	5
**AI Tools**	Use of AI to support keyword generation or search (ChatGPT, neural networks).	Traditional manual searches.	“Keyword generation via ChatGPT” (DE-167); “Neural networks and browser-based tools” (UA-501)	1	1	0	2
**Heuristic / Reflective Search**	Critical comparison and evaluation of multiple sources; precise search formulation.	Simple, one-off searches.	“Comparing info from Ukrainian and international sources, critically evaluate all” (UA-374)	0	3	0	3
**Precise Query Formulation**	Emphasis on specificity, clear wording, and defined search terms.	General or vague search terms.	“Specificity, clarity, correct wording of the request” (UA-392)	0	3	0	3
**Professional / Evidence-Based Resources**	Use of evidence-based literature, medical protocols, European clinical guidelines.	General academic or lay sources.	“Medical protocols, research articles, and specialized literature” (UA-658)	0	2	0	2
**Advanced Search Techniques**	Semantic search, truncated or wildcard terms, keyword optimization.	Simple keyword search without modifiers.	“Semantic search, truncated and wildcard searches” (UA-471)	0	2	0	2
**OSINT**	Explicit mention of open-source intelligence techniques.	General online search not labeled as OSINT.	“OSINT” (KZ-348)	0	0	1	1
**Pragmatic / Adaptive Search**	Flexible, trial-and-error approach; starting broad and refining iteratively.	Systematic, pre-planned searches.	“Firstly say something related… if I don’t find anything, I narrow it down” (KZ-491)	0	0	1	1
